# Inhibition of TrkB kinase activity impairs autophagy in cervical motor neurons of young but not old mice

**DOI:** 10.1113/EP092095

**Published:** 2024-11-22

**Authors:** Miguel Pareja‐Cajiao, Heather M. Gransee, Sepideh Jahanian, Gary C. Sieck, Carlos B. Mantilla

**Affiliations:** ^1^ Department of Anesthesiology & Perioperative Medicine Mayo Clinic Rochester Minnesota USA; ^2^ Physiology & Biomedical Engineering Mayo Clinic Rochester Minnesota USA

**Keywords:** ageing, autophagy, motor neuron, neurotrophins

## Abstract

Ageing‐related neuromuscular dysfunction is associated with reduced tropomyosin‐related kinase receptor subtype B (TrkB) signalling and accumulation of damaged cytoplasmic aggregates in motor neurons. Autophagy functions to remove these damaged aggregates, and we previously reported increased cervical motor neuron expression of LC3 and p62 in old age. We hypothesized that inhibition of TrkB kinase activity results in an increase in the relative expression of both LC3 and p62 in cervical motor neurons, consistent with impaired progression of autophagy. *TrkB^F616A^* mice, which possess a mutation that renders TrkB kinase activity susceptible to rapid inhibition by 1NMPP1, were treated at 6, 18 or 24 months of age with vehicle or 1NMPP1 for 7 days. Immunofluorescence intensity was measured to determine LC3 and p62 expression in choline acetyltransferase‐positive motor neurons in the cervical spinal cord. The effect of inhibiting TrkB kinase activity on progression of autophagy was age dependent. In 6‐month‐old mice, inhibiting TrkB kinase activity increased cervical motor neuron expression of LC3 by 11% (*P* < 0.001) and p62 by 8% (*P* = 0.019) compared with vehicle treatment. In 18‐ and 24‐month‐old mice, there was no effect of inhibiting TrkB kinase activity on motor neuron LC3 or p62 expression. We provide evidence that inhibition of TrkB signalling impairs progression of autophagy in motor neurons of young mice, similar to the response to ageing. Accordingly, a reduction of TrkB signalling in old age might contribute to neuromuscular dysfunction by impairing progression of autophagy in motor neurons.

## Abstract (highlights)

- **What is the central question of this study?**
  Ageing‐related neuromuscular dysfunction is associated with reduced tropomyosin‐related kinase receptor subtype B (TrkB) signalling and impaired progression of autophagy. The effect of inhibition of TrkB kinase activity on the progression of autophagy in motor neurons across age is unclear.
- **What is the main finding and its importance?**
  We show that inhibiting TrkB kinase activity impairs progression of autophagy in cervical motor neurons of young *TrkB^F616A^* mice, similar to the response to ageing. A reduction of TrkB signalling in old age might contribute to neuromuscular dysfunction by impairing progression of autophagy in motor neurons.

## 1 INTRODUCTION

Age‐related neuromuscular dysfunction, reflecting alterations in motor neurons, neuromuscular junctions or muscle fibres, leads to decline in muscle function. Ventilatory impairments increase in old age, and respiratory complications constitute a major cause of death in the elderly population (Heron, 2017; Houston et al., 1997; Tolep et al., 1995). Ageing of the phrenic motor system includes impairments in the progression of autophagy (Jahanian et al., 2024; Pareja‐Cajiao, Gransee, Stowe, et al., 2021), with subsequent loss of phrenic motor neurons (Fogarty et al., 2018), increased neuromuscular transmission failure (Fogarty et al., 2019; Greising, Ermilov, Sieck, et al., 2015), increased denervation of diaphragm muscle neuromuscular junctions (Greising, Stowe, Sieck, et al., 2015), reduced maximal transdiaphragmatic pressure generation (Greising, Mantilla, Medina‐Martinez, et al., 2015; Khurram et al., 2018), and diaphragm muscle sarcopenia (Elliott et al., 2016; Fogarty et al., 2020; Greising et al., 2013, Greising, Mantilla, Medina‐Martinez, et al., 2015; Khurram et al., 2018; Vang et al., 2020). Previous studies have demonstrated that signalling through the high‐affinity neurotrophin receptor tropomyosin‐related kinase receptor B (TrkB) is critical in maintaining neuromuscular function in the phrenic motor system (Mantilla et al., 2004; Mantilla & Sieck, 2008; Pareja‐Cajiao et al., 2020). These studies used *TrkB^F616A^* mice, which harbour a point mutation that renders TrkB kinase activity susceptible to rapid and selective inhibition by 1NMPP1 (Chen et al., 2005). Inhibition of TrkB kinase activity in young *TrkB^F616A^* mice (6 months of age; 100% survival) results in worsening of neuromuscular transmission failure (Mantilla et al., 2014) and impaired maximal transdiaphragmatic pressure generation (Pareja‐Cajiao, Gransee, Sieck, et al., 2021), mimicking ageing‐related effects. Inhibition of TrkB kinase activity in older *TrkB^F616A^* mice (18 and 24 months of age; 90% and 75% survival, respectively), however, fails to amplify further the ageing‐related effects on neuromuscular transmission failure (Greising, Ermilov, Sieck, et al., 2015), consistent with reduced TrkB signalling in old age.

**FIGURE 1:**
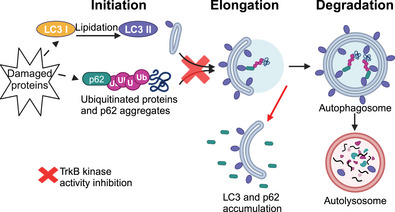
Autophagy comprises a series of evolutionarily conserved steps by which cells target damaged intracellular components for degradation in lysosomes. Initiation of autophagy culminates in the incorporation of lipidated LC3 (LC3 II) into phagophores, which elongate around damaged organelles and ubiquitinated proteins via interactions with p62. Degradation of the autophagosome and all its contents occurs after the mature autophagic vacuole membrane envelopes its cargo and is then delivered to the lysosome, where all its contents are degraded. Previous studies report contrasting effects of TrkB signalling on the autophagy markers LC3 and p62. We hypothesized that inhibition of TrkB kinase activity results in an increase in the relative expression of both LC3 and p62 in cervical motor neurons, consistent with impaired progression of autophagy (red cross and arrow). Figure created with BioRender.com.

Ageing is associated with aggregation of damaged cytoplasmic structures in motor neurons (Mattson & Magnus, 2006; Mrak et al., 1997), interfering with essential cell repair and other functions (Stead et al., 2019). Autophagy is a highly dynamic and regulated process responsible for the tagging, envelopment and recycling of damaged cellular components (Klionsky et al., 2021). Assessing the progression of autophagy requires evaluation of its several steps (Figure 1). In motor neurons, expression of the microtubule‐associated protein light chain 3 (LC3) and clearance of the ubiquitin‐tagging protein p62 have been used as indicators of elongation and degradation steps of autophagy, respectively (Pareja‐Cajiao, Gransee, Stowe, et al., 2021). Indeed, cervical and lumbar motor neurons in mice show increased relative expression of both LC3 and p62 by 24 months of age compared with 6 months (Jahanian et al., 2024; Pareja‐Cajiao, Gransee, Stowe, et al., 2021). Several previous studies have explored the effect of TrkB signalling on progression of autophagy, with contrasting results. In hippocampal neurons, TrkB signalling reduced autophagy as assessed by decreased expression of LC3 and increased expression of p62, an effect mediated through activation of the PI3K/Akt pathway (Nikoletopoulou et al., 2017). TrkB activation is also important for progression of autophagy because phosphorylation of p62, probably via the downstream TrkB target ULK1 (Lim et al., 2015), aids in the autophagosomal engulfment of cellular organelles and ubiquitinated proteins targeted for degradation (Matsumoto et al., 2011, 2015). The TrkB agonist 7,8‐dihydroflavone restored progression of autophagy in a preclinical model of Parkinson's disease (Zuo et al., 2021), and the TrkB antagonist K252a decreased autophagosome formation in rat hippocampus (Liu et al., 2020). Based on our prior results of impaired progression of autophagy in older animals and the reduced TrkB signalling in older mice, we hypothesized that inhibition of TrkB kinase activity results in an increase in the relative expression of both LC3 and p62 in cervical motor neurons.

## 2 MATERIALS AND METHODS

### 2.1 Ethical approval

All experiments were approved by the Mayo Clinic Institutional Animal Care and Use Committee (approval number A00003349), which uses the *Guide for the Care and Use of Laboratory Animals* as the reference for protocol reviews (National Research Council (US) Committee for the Update of the Guide for the Care and Use of Laboratory Animals, 2011). Additionally, we followed the principles and regulations for animal experiments outlined by Grundy (2015). All necessary procedures were implemented to minimize the pain and suffering of animals. All animals were housed in a vivarium with a 12 h–12 h light–dark cycle, with ad libitum access to water and standard laboratory chow.

### 2.2 Animals

Experiments used adult male and female *TrkB^F616A^* mice on a C57BL/6 × 129 background at 6 (*n* = 24), 18 (*n* = 12) and 24 (*n* = 12) months of age (reflecting 100%, 90% and 75% survival, respectively, based on data from our colony; Greising, Ermilov, Sieck, et al., 2015). These mice harbour a point mutation that allows for inhibition of TrkB signalling by rendering the kinase domain of TrkB receptor susceptible to robust, rapid and selective inhibition by 1‐(1,1‐dimethylethyl)‐3‐(1‐naphthalenylmethyl)‐1*H*‐pyrazolo(3,4‐d)pyrimidin‐4‐amine (1NMPP1) (Chen et al., 2005; Greising, Stowe, Sieck, et al., 2015; Mantilla et al., 2014). Animals were housed at Mayo Clinic, group caged by sex and age, and maintained in a 12 h–12 h light–dark cycle, with free access to food and water. Equal numbers of male and female mice were randomly assigned to receive 30 µL of 25 µM 1NMPP1 (MilliporeSigma; Burlington, MA, USA) in 0.3% DMSO (MilliporeSigma) or 30 µL of 0.3% DMSO in 12 mL of drinking water daily for 7 days, as in previous studies (Greising, Stowe, Sieck, et al., 2015; Mantilla et al., 2014). A subset of untreated animals at 6 months of age (*n* = 4 males and 4 females) was used to control for possible effects of DMSO; all experimental conditions were maintained, but no treatment was added to the drinking water.

A separate group of animals was used to validate the measurements of LC3 expression by removing possible effects of LC3 synthesis or degradation on the subsequent correlation between LC3 and p62 levels in individual motor neurons across all motor neuron pools. Male mice [*n* = 10; 7 (1) months] were randomly assigned to receive 1NMPP1 (*n* = 5) or vehicle (*n* = 5) in drinking water daily for 7 days, as detailed above. Animals from each group were treated with the lysosomal inhibitor chloroquine (*n* = 6; 3 1NMPP1 + chloroquine and 3 vehicle + chloroquine; 50 mg/kg via intraperitoneal injection) or vehicle (*n* = 4; 2 1NMPP1 + vehicle and 2 vehicle + vehicle) for 4 h before the terminal experiment (Gurney et al., 2015; Klionsky et al., 2021). The median relative LC3 expression across all motor neurons in each animal was obtained, and the median relative LC3 expression across all animals in each group was reported. An LC3 synthesis ratio was then calculated as the ratio of the difference between the median LC3 expression in the 1NMPP1 + chloroquine and the vehicle + saline groups divided by the difference in the median LC3 expression in the vehicle + chloroquine and vehicle + saline groups. Increased LC3, resulting from greater initiation of autophagy (LC3 synthesis ratio > 1), can thus be distinguished from increases in LC3 resulting from impaired autophagosome degradation (LC3 synthesis ratio < 1) (Klionsky et al., 2021). These changes in LC3 expression can then be correlated with changes in p62 levels to assess the progression of autophagy.

At the terminal experiment, mice were weighed, anaesthetized by intraperitoneal injection of fentanyl (0.3 mg/kg; Hospira, Inc.; Lake Forest, IL, USA), diazepam (5 mg/kg; DASH Pharmaceuticals LLC; Upper Saddle River, NJ, USA) and droperidol (15 mg/kg; American Regent, Inc; Shirley, NY, USA), and euthanized by exsanguination. Animals were then perfused transcardially with 4% paraformaldehyde, and the cervical spinal cord was harvested and processed for subsequent immunofluorescence analyses.

### 2.3 Immunofluorescence processing

As previously described (Jahanian et al., 2024; Pareja‐Cajiao, Gransee, Stowe, et al., 2021), the cervical spinal cord was resected, placed in 4% paraformaldehyde overnight, then transferred to 30% sucrose at 4°C. Spinal cords were assembled in ‘batches’ such that all treatment groups could be sectioned simultaneously. For the 6‐month‐old animal batches, six spinal cords were embedded together, including one male and one female animal from each treatment group (control, vehicle and 1NMPP1); for the 18‐ and 24‐month‐old animal batches, four spinal cords from same‐sex animals were embedded together, including one animal from each age (18 and 24 months) and treatment (vehicle or 1NMPP1) group. This design was later considered for image analyses (see below). Each ‘batch’ was then sectioned at 20 µm thickness in the transverse plane and mounted directly onto Superfrost Plus slides (Fisher Scientific, Pittsburgh, PA, USA). Consecutive sections were placed in separate slides, such that adjacent sections within a slide were 200 µm apart. This stereological approach was used to generate 10 equally spaced ‘sets’ representing the entirety of the cervical spinal cord. Slides were kept at −80°C until further processing.

A single set of slides containing ∼35 sections per animal was selected for immunofluorescence using a random number generator. Sections were washed in TBS followed by antigen retrieval in 10 mM tri‐sodium citrate containing 0.05% Tween 20 at 80°C for 30 min. After blocking in 10% donkey serum in 0.3% Triton X‐100–TBS, they were incubated with primary antibodies (all in 5% donkey serum in 0.3% Triton X‐100–TBS) for LC3B (NB100‐2220, rabbit; 1:200; Novus Biologicals, Centennial, CO, USA), p62 (GPg2‐C, guinea pig; 1:100; Progen, Wayne, PA, USA) and choline acetyltransferase (ChAT, AB144P, goat; 1:200; Millipore, Burlington, MA, USA) at 4°C. After washes, sections were incubated in corresponding Alexa Fluor‐conjugated secondary antibodies from Jackson ImmunoResearch [Alexa Fluor 647 donkey anti‐rabbit (711‐605‐152), Alexa Fluor 594 donkey anti‐guinea pig (706‐585‐148) and Alexa Fluor 488 donkey anti‐goat (705‐545‐147)]. Sections were treated with ProLong™ Gold containing 4′,6‐diamidino‐2‐phenylindole (Thermo Fischer) and coverslipped.

### 2.4 Confocal microscopy

**FIGURE 2:**
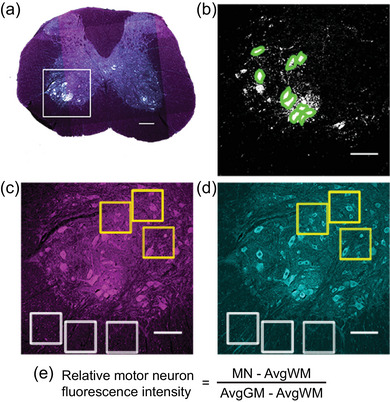
Immunohistochemical measurements of LC3 and p62 expression in motor neurons from spinal cord segments C3–C5 followed the methods described previously (Pareja‐Cajiao, Gransee, Stowe, et al., 2021). (a) Briefly, single‐slice images of the spinal cord taken with the 10× objective were stitched and used to identify the spinal segment referencing a standard atlas. Multicoloured labelling shows that the grey matter displays predominant fluorescence of ChAT (white), LC3 (magenta) and p62 (cyan). The inset in (a) represents the area used to obtain a 20× image for immunofluorescence intensity analyses in (b–d). (b) Motor neurons were identified by ChAT reactivity, and a ROI was hand‐drawn around each motor neuron (green outline) that had a mid‐nuclear section in the *z*‐stack. (c and d) Six ROIs of constant shape and area were placed in white matter and grey matter indicated by white and yellow inlays, respectively. (e) Formula used for analyses of relative motor neuron fluorescence intensity. Scale bars: 200 µm (a) and 100 µm (b–d). Abbreviations: AvgGM, average of the grey matter intensity for the three ROIs in the image; AvgWM, average of white matter intensity for the three white matter ROIs in the same image; ChAT, choline acetyltransferase; MN, motor neuron intensity; ROI, region of interest.

Immunofluorescent labelling was detected using an Olympus FluoView FV1200 laser scanning confocal system (Olympus America Inc., Melville, NY, USA) with four diode lasers (405, 488, 559 and 635 nm) and four fluorescence detectors mounted on an Olympus BX61 microscope. Individual images obtained using the 10× objective (NA 0.3) were stitched to evaluate grey matter anatomy and determine spinal cord segment (Figure 2a) using a standard atlas (Sengul et al., 2012); no intensity analyses were performed on the 10× magnification images. Sections from segments C3–C5 were imaged using an oil‐immersion 20× objective (NA 0.8). Image stacks (12‐bit, 1024 pixels × 1024 pixels, 2 µm step size) containing ChAT‐positive neurons were obtained of the ventral horn on each side of the spinal cord (Figure 2c). The number of confocal slices varied across images. To account for possible variability in immunofluorescence intensity across slides, laser intensity, voltage and gain were set in each channel such that the average intensity of the white matter across untreated animals was ∼10%–15% of the dynamic range and the motor neurons of the 1NMPP1‐treated sample in that slide displayed ≤5% pixel saturation. All images within a slide were acquired using the same settings.

### 2.5 Image analysis

Confocal image stacks were processed using Metamorph (Molecular Devices, San Jose, CA, USA) and NIS‐Elements (Nikon, Melville, NY, USA). Images with extensive tissue damage, folds or bleaching were not included for analyses. Regions of interest (ROIs) were drawn as follows: three ROIs of constant area in both the grey matter and the white matter, excluding sites that showed micro‐tears in the tissue (Figure 2c,d). Grey matter ROIs were placed in regions of the ventral horn that did not contain ChAT‐immunoreactive cells. Motor neurons were identified as large ChAT‐immunoreactive cells with a cross‐sectional area of >200 µm^2^ and surrounded by pericellular ChAT‐immunoreactive boutons. A ROI was drawn manually around each motor neuron displaying a mid‐nuclear section in the *z*‐stack (Figure 2b).

The fluorescence channels for LC3 and p62 were analysed separately after obtaining a maximum intensity projection image. Mean fluorescence intensity was determined for every ROI (motor neuron, grey and white matter) in the ventral horn on each side of the spinal cord. Semiquantitative analyses of fluorescence intensity were facilitated by determining the variability in fluorescence intensity for the white and grey matter of the ventral spinal cord in sections representing the midcervical spinal cord enlargement (C3–C5). Accordingly, average fluorescence intensity was evaluated across sections, slides and ‘batches’ to ensure consistency of staining, imaging conditions and analyses. Based on our prior study (Pareja‐Cajiao, Gransee, Stowe, et al., 2021), white matter fluorescence was considered background, hence the average fluorescence intensity for the three white matter ROIs was subtracted from all other ROIs. To evaluate the localization of any changes in the relative LC3 and p62 fluorescence intensities (e.g., cell body vs. neuropil or other nonmotor neuron cells in the grey matter), the ratio of the mean fluorescence intensity of each motor neuron to the average of grey matter ROIs in that section was then determined (Figure 2e). Each motor neuron was identified as a putative phrenic motor neuron or as belonging to non‐phrenic motor neuron pools based on its location within the grey matter compared with a standard mouse spinal cord atlas (Sengul et al., 2012). Finally, the relative fluorescence intensity for each motor neuron was normalized to the 18‐month‐old vehicle group in each slide (or the 6‐month‐old female vehicle group in the DMSO treatment subset).

In additional analyses, the relative fluorescence intensity of LC3 was plotted versus the relative fluorescence intensity of p62 in each individual motor neuron in order to provide a visual assessment of the progression of autophagy, as in a previous study (Pareja‐Cajiao, Gransee, Stowe, et al., 2021). In these analyses per motor neuron, *z*‐scores of the relative fluorescence intensity were calculated from the overall mean and SD for all animals within each age group. After all analyses were completed and for visualization purposes only, the brightness and contrast were adjusted for all representative images simultaneously.

### 2.6 Statistical analyses

All statistical evaluations were performed using standard statistical software (JMP v.17, SAS Institute Inc., Cary, NC, USA). Animal weight was compared using a three‐way ANOVA, with sex, age, treatment and their interactions as fixed effects. A two‐way ANOVA for each age group, with treatment, sex and their interaction as fixed effects, was used to compare grey matter or white matter intensity. For each group and motor neuron pool, the relative fluorescence intensity for LC3 and p62 in each motor neuron within each slide was averaged, and then these values were averaged for each animal. The animal average relative LC3 and p62 expression was compared using a three‐way ANOVA, with treatment, sex, motor neuron pool and their interactions as fixed effects. A one‐way ANOVA, with treatment as a fixed effect, was used to compare the animal average relative LC3 and p62 in motor neurons in animals treated with DMSO, water or 1NMPP1.

Table 1 provides information on the study population, with the numbers of animals studied in each age group, sex and treatment, absolute number of motor neurons studied, and number of motor neurons per animal that were included in the analyses of both LC3 and p62 expression. Based on previous data showing a sex difference only in the young animals (Pareja‐Cajiao, Gransee, Stowe, et al., 2021), we estimated that eight animals per group would be sufficient to detect a 20%–25% difference in both proteins across sex groups, with 80% power and α = 0.05. For the aged animals, we estimated that six animals per group would be sufficient to detect a 20% change in relative expression of both proteins after treatment, with a power of 80% and α = 0.05. When appropriate, *post hoc* analyses were conducted using a Tukey–Kramer HSD test. In all cases, statistical significance was established at *P* < 0.050. Data are presented in arbitrary units, with the relative fluorescence intensity data compared with the white matter‐subtracted grey matter value for each motor neuron. All the experimental data in this text are presented as the mean (SD), unless otherwise specified.

**TABLE 1:** Study population.

				Per animal		
Age (months)	Treatment	Animals [total (male, female)]	Total motor neurons [total (male, female)]	Phrenic motor neurons [median (minimum, maximum)]	Non‐phrenic motor neurons [median (minimum, maximum)]	Total motor neurons [median (minimum, maximum)]
6	Vehicle	8 (4, 4)	811 (369, 442)	30 (18, 42)	69 (59, 96)	99 (77, 133)
	1NMPP1	8 (4, 4)	677 (255, 422)	24 (21, 34)	53 (33, 88)	77 (54, 121)
18	Vehicle	6 (3, 3)	708 (348, 360)	31 (17, 37)	89 (79, 102)	119 (103, 139)
	1NMPP1	6 (3, 3)	466 (261, 205)	22 (10, 56)	51 (33, 78)	77 (51, 102)
24	Vehicle	5 (3, 2)	399 (234, 165)	27 (14, 32)	64 (27, 71)	94 (42, 98)
	1NMPP1	6 (3, 3)	583 (254, 329)	23 (12, 36)	68 (31, 97)	85 (45, 131)

*Note*: Numbers of animals studied in each age group, sex and treatment, absolute number of motor neurons studied, and number of motor neurons per animal [median [minimum, maximum)] that were included in the analyses of both LC3 and p62 expression are reported. Motor neurons in the cervical spinal cord were classified as phrenic and non‐phrenic neurons anatomically (see Materials and Methods).

## 3 RESULTS

### 3.1 Animals

A total of 48 *TrkB^F616A^* mice equally balanced by sex were used in the experiments. There was a significant effect on body weight of sex (*F*_1,36_ = 44; *P* < 0.001), but not of age (*F*_2,36 _= 2; *P* = 0.201) or treatment (*F*_1,36 _< 1; *P* = 0.470). There was no age × sex (*F*_2,36 _< 1; *P* = 0.427), age × treatment (*F*_2,36 _< 1; *P* = 0.640), sex × treatment (*F*_2,36 _< 1; *P* = 0.999) or age × treatment × sex (*F*_2,36 _< 1; *P* = 0.700) interaction. The average weight was 30.5 (1.3), 29.5 (3.0) and 31.8 (2.6) g for the 6‐, 18‐ and 24‐month‐old groups, respectively. *Post hoc* analyses revealed that male mice were of significantly greater weight than females of the same age group for the 6‐month [33.6 (2.0) vs. 27.6 (3.8) g, respectively; *P* < 0.050] and 18‐month‐old groups [33.6 (2.2) vs. 25.4 (2.4) g, respectively; *P* < 0.050], but this difference was no longer significant by 24 months of age [34.4 (2.4) vs. 29.3 (4.0) g, respectively].

### 3.2 Imaging settings did not affect grey or white matter intensity across images within groups

The average fluorescence intensity for grey matter and white matter varied across sections within age groups. At 6 months, the average grey matter intensity for LC3 and p62 was 1549 (1350, 1961) [median (minimum, maximum)] and 900 (765, 1177) a.u., respectively; and the white matter intensity for LC3 and p62 was 981 (882, 1182) and 519 (477, 662) a.u., respectively. At 18 months, the grey matter intensity for LC3 and p62 was 1465 (1104, 2028) and 1277 (1077, 1682) a.u., respectively; and the white matter intensity for LC3 and p62 was 772 (541, 1149) and 697 (524, 880) a.u., respectively. At 24 months, the grey matter intensity for LC3 and p62 was 1372 (1098, 1840) and 1139 (1012, 1536) a.u., respectively; and the white matter intensity for LC3 and p62 was 751 (563, 1035) and 633 (548, 852) a.u., respectively. A two‐way ANOVA for each age group, with treatment, sex and their interaction, did not show any effects on the average grey matter or white matter intensity (*P* > 0.050 in all cases).

Taken together, these findings suggest that the slight differences in the imaging settings were not a significant source of variability for the grey matter and white matter fluorescence intensity for animals within each age group, hence these results provide confidence that the outcome of interest (relative LC3 or p62 fluorescence intensity for each animal) was comparable across slides and ‘batches’.

### 3.3 Inhibition of TrkB kinase activity results in LC3 accumulation in young mice

**FIGURE 3:**
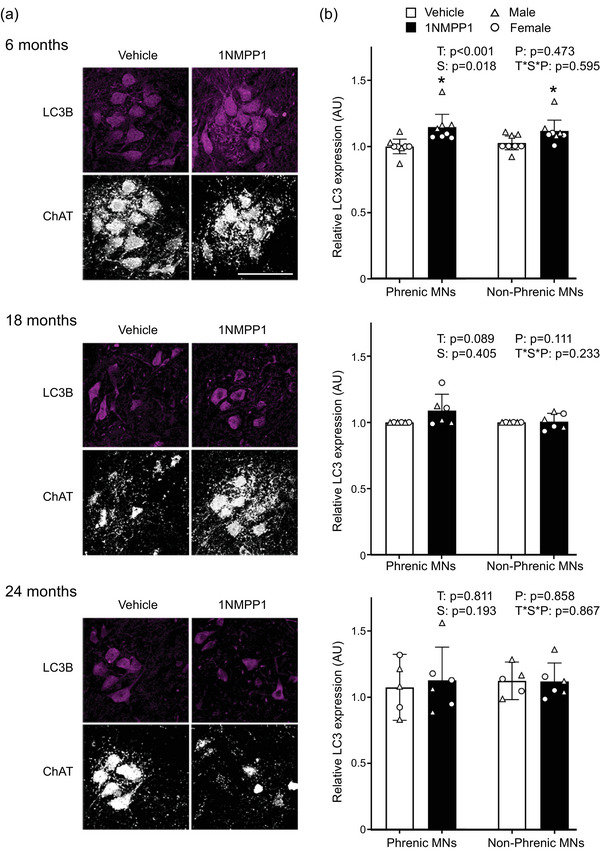
Expression of LC3 in motor neurons of mice across age groups after inhibition of TrkB kinase activity. (a) Maximum intensity projection images for LC3 and ChAT immunofluorescence in motor neurons of mice at 6, 18 and 24 months of age. (b) Bar graphs show mean ± 95% confidence interval of LC3 expression in putative phrenic and non‐phrenic MNs, averaged per animal. Number of animals: 6 months, *n* = 4 males and 4 females for both treatments and motor neuron pools; 18 months, *n* = 3 males and 3 females for both treatments and motor neuron pools; 24 months, *n* = 3 males and 2 females for vehicle treatment and both motor neuron pools; *n* = 3 males and 3 females for 1NMPP1 treatment and both motor neuron pools. Scale bar: 100 µm. ^*^Overall effect of 1NMPP1 compared with vehicle group. Abbreviations: MNs, motor neurons; P, motor neuron pool; S, sex; T, treatment; T*S*P, treatment × sex × motor neuron pool interaction.

In order to evaluate the effects of inhibiting TrkB kinase activity on progression of autophagy, motor neuron expression of LC3 relative to grey matter (relative LC3) was measured as an indicator of the amount of phagophore and autophagosome within cells (Figure 3) (Klionsky et al., 2021). At 6 months of age, there was an effect on relative LC3 expression of treatment (*F*_1,1 _= 15; *P* < 0.001) and sex (*F*_1,1 _= 7; *P* = 0.018), but not of motor neuron pool (*F*_1,1 _< 1; *P* = 0.473) or the three‐way interaction (*F*_1,1 _< 1; *P* = 0.595). Inhibiton of TrkB kinase activity with 1NMPP1 increased LC3 relative expression by 11% compared with vehicle treatment when both sex and phrenic/non‐phrenic motor neurons were pooled. Males across both treatment groups and motor neuron pools displayed 7% higher LC3 relative expression than females. *Post hoc* analyses revealed that 1NMPP1 treatment in male mice significantly increased relative LC3 expression in phrenic motor neurons by 18% compared with vehicle treatment (*P* < 0.050). 1NMPP1 did not change relative LC3 expression in phrenic motor neurons from female mice or in non‐phrenic motor neurons.

At 18 months of age, there was no effect on LC3 expression of treatment (*F*_1,1 _= 3; *P* = 0.089), sex (*F*_1,1 _< 1; *P* = 0.405), motor neuron pool (*F*_1,1 _= 3; *P* = 0.111) or the three‐way interaction (*F*_1,1 _= 2; *P* = 0.233). Likewise, at 24 months of age, there was no effect on LC3 expression of treatment (*F*_1,1 _< 1; *P* = 0.811), sex (*F*_1,1 _= 2; *P* = 0.193), motor neuron pool (*F*_1,1 _< 1; *P* = 0.858) or the three‐way interaction (*F*_1,1 _< 1; *P* = 0.866).

### 3.4 Inhibition of TrkB kinase activity results in p62 accumulation in young mice

**FIGURE 4:**
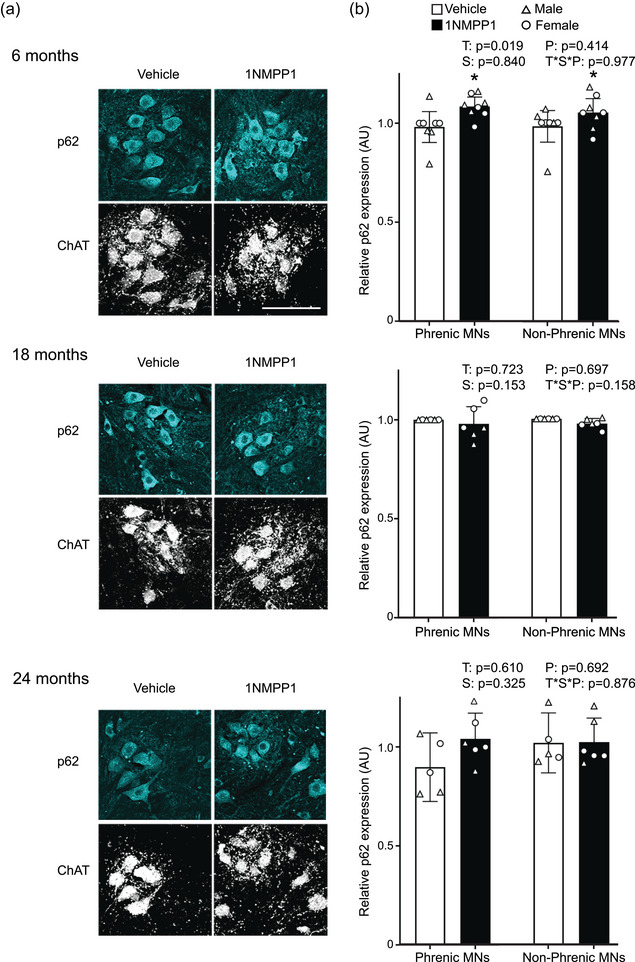
Expression of p62 in motor neurons of mice across age groups after inhibition of TrkB kinase activity. (a) Maximum intensity projection images for p62 and ChAT immunofluorescence in motor neurons of mice at 6, 18 and 24 months of age. (b) Bar graphs show mean ± 95% confidence interval of p62 expression in putative phrenic and non‐phrenic MNs, averaged per animal. Number of animals: 6 months, *n* = 4 males and 4 females for both treatments and motor neuron pools; 18 months, *n* = 3 males and 3 females for both treatments and motor neuron pools; 24 months, *n* = 3 males and 2 females for vehicle treatment and both motor neuron pools; *n* = 3 males and 3 females for 1NMPP1 treatment and both motor neuron pools. Scale bar: 100 µm. ^*^Overall effect of 1NMPP1 compared with vehicle group. Abbreviations: MNs, motor neurons; P, motor neuron pool; S, sex; T, treatment; T*S*P, treatment × sex × motor neuron pool interaction.

When the ubiquitin‐binding protein p62 interacts with LC3, the autophagosome membrane elongates around the p62‐labelled cargoes; then the complete autophagosomes are delivered to lysosomes for degradation. Thus, clearance of p62 can be used as a marker of the degradation step of autophagy. At 6 months of age, there was an effect on relative p62 expression of treatment (*F*_1,1 _= 6; *P* = 0.019) but not of sex (*F*_1,1 _< 1; *P* = 0.840), motor neuron pool (*F*_1,1 _< 1; *P* = 0.414) or the three‐way interaction (*F*_1,1 _< 1; *P* = 0.977; Figure 4). 1NMPP1 treatment increased relative p62 expression by 8% compared with vehicle treatment at 6 months of age when both sex and phrenic/non‐phrenic motor neurons were pooled.

At 18 months of age, there was no effect on p62 expression of treatment (*F*_1,1 _< 1; *P* = 0.723), sex (*F*_1,1 _= 2; *P* = 0.153), motor neuron pool (*F*_1,1 _< 1; *P* = 0.697) or the three‐way interaction (*F*_1,1 _= 2; *P* = 0.158). Likewise, at 24 months of age, there was no effect on p62 expression of treatment (*F*_1,1 _< 1; *P* = 0.610), sex (*F*_1,1 _= 1; *P* = 0.325), motor neuron pool (*F*_1,1 _< 1; *P* = 0.692) or the three‐way interaction (*F*_1,1 _< 1; *P* = 0.876).

### 3.5 Vehicle (DMSO) treatment does not change LC3 or p62 expression

A group of untreated 6‐month‐old mice (*n* = 4 male and 4 female) were compared with 6‐month‐old mice treated with 0.3% DMSO (*n* = 4 male and 4 female) or 25 µM 1NMPP1 in 0.3% DMSO (*n* = 4 male and 4 female) in order to assess possible effects of the DMSO in the vehicle‐treated animals. There was an overall effect on LC3 expression of treatment (*F*_2,23 _= 9; *P* = 0.001). *Post hoc* analyses showed that 1NMPP1 treatment significantly increased LC3 [2.37 (0.22) a.u.] compared with DMSO [2.15 (0.10) a.u.; *P* = 0.028] or water [2.04 (0.13) a.u.; *P* = 0.001] treatment. There was no significant difference in LC3 expression between DMSO and water treatment (*P* = 0.345). Likewise, there was an overall effect on p62 expression of treatment (*F*_2,23 _= 6; *P* < 0.009). *Post hoc* analyses showed that 1NMPP1 treatment significantly increased p62 [2.44 (0.18) a.u.] compared with water treatment [2.19 (0.11) a.u.; *P* = 0.009]. There was no significant difference in p62 between DMSO [2.26 (0.15) a.u.] and water treatment (*P* = 0.628). Overall, DMSO treatment itself did not affect relative LC3 or p62 expression in motor neurons.

### 3.6 Correlation between LC3 and p62 levels in individual motor neurons

In order to validate the measurements of LC3 expression in individual motor neurons, the possible effects of changes in LC3 synthesis or degradation were determined by inhibiting autophagosomal degradation at the terminal experiment (see Materials and Methods). In a subset of 7‐month‐old male mice with or without 1NMPP1 treatment and injected with the lysosomal inhibitor chloroquine, the median LC3 and p62 expression across all motor neurons in each treatment group was obtained (Table 2). The LC3 synthesis ratio was calculated to be 0.85, which suggests that combined 1NMPP1 and chloroquine treatment is not changing LC3 expression any more than chloroquine treatment alone. Thus, by blocking the degradation of LC3 with chloroquine, we found that the accumulation of LC3 following 1NMPP1 treatment does not reflect increased LC3 synthesis (ratio < 1). As expected, p62 expression in motor neurons increased with chloroquine treatment, confirming impaired autophagosome degradation.

**TABLE 2:** Relative LC3 and p62 expression in subset of animals treated with autophagy inhibitor.

TrkB inhibitor Treatment	Autophagy inhibitor Treatment	Animals	Motor neurons	Relative LC3 expression [median (minimum, maximum)] (a.u.)	Relative p62 expression [median (minimum, maximum)] (a.u.)
Vehicle	Vehicle	2	142	1.36 (1.35, 1.36)	2.36 (2.25, 2.47)
Vehicle	Chloroquine	3	183	1.49 (1.41, 1.89)	2.59 (2.50, 3.04)
1NMPP1	Vehicle	2	80	1.46 (1.32, 1.59)	2.45 (2.34, 2.55)
1NMPP1	Chloroquine	3	246	1.47 (1.33, 1.86)	2.91 (2.40, 3.02)

*Note*: Median (minimum, maximum) values for relative LC3 and p62 expression (see Figure 2 for formula) are given across all motor neurons per animal in each group of inhibitor treatments. Data represent the number of animals, the total number of motor neurons (phrenic and non‐phrenic pooled), and the median, minimum and maximum of relative LC3 and p62 values per animal in each treatment group.

**FIGURE 5:**
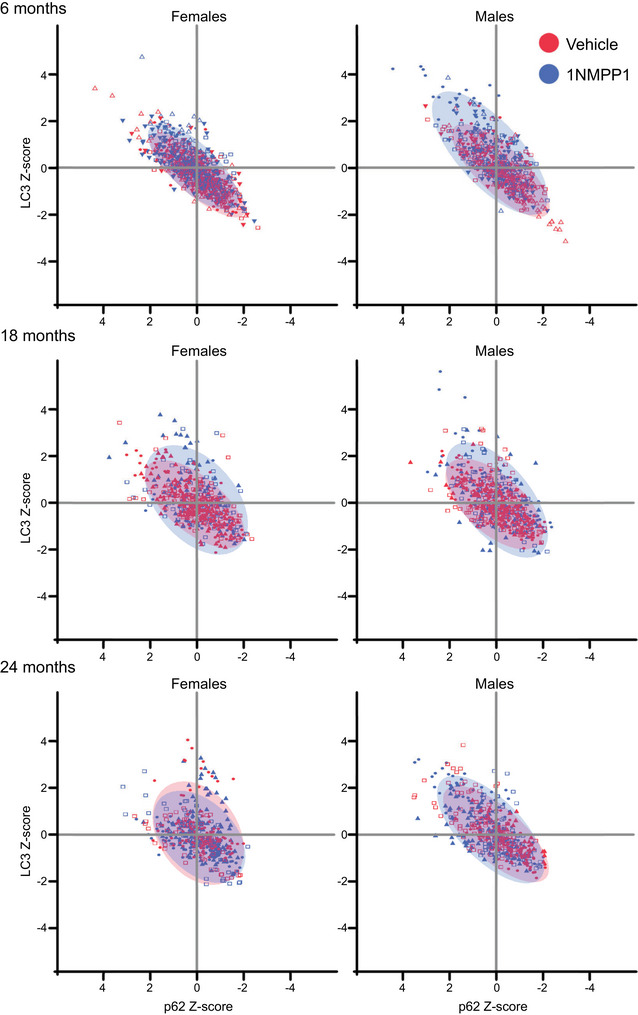
Correlation between the relative expression of LC3 and p62 in individual motor neurons of 1NMPP1‐ or control‐treated mice, separated by age and sex. Colours represent treatment groups, symbols represent animals, and each point represents the expression at an individual motor neuron. The mean and SD from all animals were used to calculate the *z*‐scores. Bivariate normal density ellipses (90% of distribution) are represented as shaded areas for each treatment group. At 6 months of age, the 1NMPP1‐treated group for both male and female mice displayed a shift to the upper left corner (impaired autophagy) when compared with the vehicle control group. 1NMPP1 treatment had minimal effects on LC3 or p62 expression in the motor neurons of aged mice at 18 or 24 months of age. Motor neuron numbers for each age, treatment and sex group are presented in Table 1.

The correlation between the relative LC3 and p62 expression in individual motor neurons can be used to evaluate visually changes in progression of autophagy (Figure 5) (Pareja‐Cajiao, Gransee, Stowe, et al., 2021). All motor neurons were plotted together to illustrate the effect of 1NMPP1 on the expression of both LC3 and p62 for each age group and sex. *Z*‐Scores were calculated from the overall mean and SD of relative LC3 and p62 fluorescence measurements, for each age group. Overall, there was a shift towards increased LC3 and p62 in motor neurons in the cervical spinal cord of 6‐month‐old mice after 1NMPP1 treatment compared with vehicle, consistent with impaired progression of autophagy. This shift towards increased LC3 and p62 was most visible in the 6‐month‐old male mice. The shift towards impaired autophagy was not evident in 18‐ or 24‐month‐old mice.

## 4 DISCUSSION

Our present results show that inhibition of TrkB kinase activity impairs progression of autophagy at motor neurons in the cervical spinal cord of *TrkB^F616A^* mice at 6 months of age, but this effect is no longer significant in mice at 18 or 24 months of age. In young *TrkB^F616A^* mice, inhibition of TrkB kinase activity increased immunoreactivity for both LC3 and p62 in motor neurons of the cervical spinal cord, reflecting an impairment of the progression of autophagy and mimicking the ageing‐related effects previously described (Pareja‐Cajiao, Gransee, Stowe, et al., 2021). We found additional evidence suggesting that the effect of TrkB kinase inhibition on the progression of autophagy in young mice differs across sexes, and lower LC3 levels in females suggest reduced phagophores. The increased p62 expression in cervical motor neurons in young male and female mice suggests autophagosome accumulation. The 1NMPP1 treatment and sex differences were no longer present in mice at 18 or 24 months of age. Notably, cervical (and also lumbar) motor neurons in mice show increased relative expression of both LC3 and p62 by 24 months compared with 6 months of age (Jahanian et al., 2024; Pareja‐Cajiao, Gransee, Stowe, et al., 2021). Taken together, these results provide evidence that TrkB kinase activity is important in maintaining the delicate balance of autophagy required for cellular homeostasis in the cervical motor neurons of young mice and that the ageing‐related loss of TrkB kinase activity might contribute to impairment of autophagy in addition to neuromuscular dysfunction in old age.

Ageing comprises several processes, including multisystemic abnormalities that culminate in physiological dysfunction and increased vulnerability to death (Lopez‐Otin et al., 2013). Indeed, ageing of the phrenic motor system is characterized by a series of alterations that initiate at the motor neuron and progress to dysfunction of the diaphragm muscle. The ageing‐related effects start early in old age but are evident well before the effects on diaphragm muscle specific force or atrophy appear (Greising, Mantilla, Medina‐Martinez, et al., 2015; Greising et al., 2017). In old age, there is a ∼30% increase in neuromuscular transmission failure (Greising, Ermilov, Sieck, et al., 2015), a ∼20% increase in denervation of diaphragm fibres (Greising, Stowe, Sieck, et al., 2015), a ∼30% reduction of maximal transdiaphragmatic pressure generation (Greising, Mantilla, Medina‐Martinez, et al., 2015; Pareja‐Cajiao, Gransee, Sieck, et al., 2021) and a loss of ∼20% of phrenic motor neurons (Fogarty et al., 2018). LC3 has been shown to accumulate in the cervical motor neurons of mice by 24 months of age when compared with 6 and 18 months of age, with small differences across phrenic versus non‐phrenic motor neuron pools (Pareja‐Cajiao, Gransee, Stowe, et al., 2021). Clearance of p62 in motor neurons has been shown to be reduced in cervical motor neurons by 18 months compared with 6 months of age, with no further difference by 24 months of age (Pareja‐Cajiao, Gransee, Stowe, et al., 2021). Accumulation of autophagosomes, reflected by an increase of ∼20% and ∼30% in motor neuron expression of LC3 and p62, respectively, suggests progressive impairment of autophagy in old age (Pareja‐Cajiao, Gransee, Stowe, et al., 2021). Interestingly, the present study shows that inhibition of TrkB kinase activity causes similar effects on LC3 and p62 expression in cervical motor neurons in 6‐month‐old mice. Of note, the present study focused on the effect of 1NMPP1 on LC3 and p62 expression in cervical motor neurons at each age group. The experimental design facilitated analysis of this treatment effect, but not of the age effect on autophagy protein expression that we have previously shown (Pareja‐Cajiao, Gransee, Stowe, et al., 2021).

Of the cellular and molecular hallmarks of ageing, impairment in progression of autophagy is of particular importance. Autophagy is a highly dynamic and regulated process responsible for the tagging, envelopment and recycling of damaged cellular components that might contribute to coping with accumulation of damaged cytoplasmic structures, such as proteins or organelles (Lopez‐Otin et al., 2013). In postmitotic cells, such as motor neurons, this accumulation is not mitigated by cell division (Ashrafi et al., 2014; Loos et al., 2017; Rubinsztein et al., 2011; Stead et al., 2019), thus stressing the importance of balancing autophagy into old age. Indeed, there is an ageing‐related impairment of autophagy across several tissues and organisms, including the CNS of mice (Hansen et al., 2018; Komatsu et al., 2006), cervical and lumbar motor neurons (Jahanian et al., 2024; Pareja‐Cajiao, Gransee, Stowe, et al., 2021) and, specifically, putative phrenic motor neurons (Pareja‐Cajiao, Gransee, Stowe, et al., 2021).

Signalling through the TrkB receptor in the phrenic motor system is crucial to sustaining neuromuscular transmission (Greising, Stowe, Sieck, et al., 2015; Mantilla et al., 2004, 2008), and generating maximal forces by the diaphragm muscle (Pareja‐Cajiao et al., 2020). Indeed, inhibition of TrkB kinase activity in young *TrkB^F616A^* mice reproduces the ageing‐related impairments in neuromuscular function, including worsening of neuromuscular transmission failure (Greising, Ermilov, Sieck, et al., 2015) and reduced maximal transdiaphragmatic pressure generation (Pareja‐Cajiao et al., 2020). However, a series of prior studies show that the role of TrkB in sustaining neuromuscular function at the phrenic motor system declines with age. In 18‐month‐old *TrkB^F616A^* mice (90% survival), treatment with TrkB agonists improved neuromuscular transmission compared with the untreated group (Greising, Ermilov, Sieck, et al., 2015), whereas inhibition of TrkB kinase activity impaired the ability of the diaphragm muscle to generate maximal transdiaphragmatic pressure (Pareja‐Cajiao, Gransee, Sieck, et al., 2021). By 24 months of age (75% survival), neither treatment with TrkB agonists nor inhibition of TrkB kinase activity modified the ageing‐related effects on neuromuscular function at the phrenic motor system (Greising et al., 2017). This loss of TrkB signalling in old age might reflect changes in receptor expression or downstream effects that remain to be elucidated.

We previously reported a sex‐based difference in the basal levels of autophagy in cervical but not lumbar motor neurons (Jahanian et al., 2024; Pareja‐Cajiao, Gransee, Stowe, et al., 2021). Cervical motor neurons from young female mice displayed ∼20% higher p62 expression than age‐matched males but no evidence of changed expression up to 24 months of age (Pareja‐Cajiao, Gransee, Stowe, et al., 2021). Notably, p62 levels increase when autophagosomes are not degraded as expected during the autophagy process (Figure 1). Our present data suggest reduced autophagy in young females at 6 months of age compared with males, and autophagosome accumulation despite reduced phagophores, as indicated by the ∼7% overall lower expression of LC3 in cervical motor neurons in females compared with males. Indeed, there is evidence that sex hormone signalling pathways interact with autophagy (Yang & Klionsky, 2009), whereby young females display reduced autophagy (Xiang et al., 2019) even in motor neurons (Olivan et al., 2014). Of note, there is no evidence of a sex‐based difference in the neuromuscular function at the phrenic motor system, nor in the response to ageing or inhibition of TrkB kinase activity (Fogarty et al., 2018; Greising, Mantilla, Medina‐Martinez, et al., 2015; Pareja‐Cajiao et al., 2020).

Recently, autophagy has become a mechanism of interest owing to its role in modulating neurotransmitter release at the presynaptic terminal of neuromuscular junctions (Birdsall & Waites, 2019; Lieberman & Sulzer, 2020). A previous study evaluating the effects of TrkB signalling on autophagy at the synapse showed that TrkB signalling reduced autophagy by decreasing expression of LC3 and increasing expression of p62 through activation of the PI3K/Akt pathway in hippocampal neurons (Nikoletopoulou et al., 2017). Their results suggest that activation of TrkB decreases the number of phagophores, possibly at steps earlier than autophagosome–lysosome fusion. In the present study, we used chloroquine to block autophagosomal degradation and found no evidence of increased LC3 synthesis following 1NMPP1 treatment. Basal levels of autophagy expression might vary across tissues and conditions, potentially confounding interpretation across these studies. Indeed, in the presence of an autophagy‐activating stressor in the hippocampus, the TrkB kinase inhibitor K252a reverses the autophagy‐activating signal and impairs autophagy by decreasing expression of the initiation factor beclin‐1 and increasing expression of p62 (Liu et al., 2020). Taken together, these studies are consistent with TrkB kinase inhibition resulting in autophagosome accumulation. It is noteworthy that phosphorylation of p62 by several serine kinases might facilitate progression of autophagy, probably by modifying the LC3–p62 interactions (Matsumoto et al., 2015, 2011). Recent work showed that activation of TrkB activates the autophagy‐initiation complex comprising ULK1 (Zuo et al., 2021), which has also been shown to phosphorylate p62 (Lim et al., 2015). These results point at a direct interaction between TrkB signalling and progression of autophagy. An ageing‐related decrease in TrkB signalling through reduction of the TrkB ligand brain‐derived neurotrophic factor or the receptor expression might contribute to the ageing‐related neuromuscular dysfunction, probably through dysregulation of autophagy.

## 5 CONCLUSION

In summary, the present study shows that inhibition of TrkB kinase activity impairs progression of autophagy only in young mice. Differences in the effects of inhibiting TrkB kinase activity across age groups point to a window of vulnerability in aged mice, probably before 18 months of age. Altogether, these data support impairment of autophagy as a key mechanism at the intersection between ageing‐related decline in TrkB signalling and neuromuscular dysfunction. Activated TrkB receptors that are retrogradely transported inside autophagosomes might serve to bolster neuronal survival, promote neuronal branching and prevent neurodegeneration (Ginty & Segal, 2002; Kononenko et al., 2017), including in old age.

## AUTHOR CONTRIBUTIONS

Conception or design of the work: Miguel Pareja‐Cajiao, Heather M. Gransee, Gary C. Sieck and Carlos B. Mantilla. Acquisition, analysis or interpretation of data for the work: Miguel Pareja‐Cajiao, Heather M. Gransee, Sepideh Jahanian, Gary C. Sieck and Carlos B. Mantilla. Drafting of the work or revising it critically for important intellectual content: Miguel Pareja‐Cajiao, Heather M. Gransee, Sepideh Jahanian, Gary C. Sieck and Carlos B. Mantilla. All authors approved the final version of the manuscript and agree to be accountable for all aspects of the work in ensuring that questions related to the accuracy or integrity of any part of the work are appropriately investigated and resolved. All persons designated as authors qualify for authorship, and all those who qualify for authorship are listed.

## CONFLICT OF INTEREST

The authors declare no conflicts of interest.

## Data Availability

The data that support the findings of this study are available from the corresponding author upon reasonable request.
